# Peritoneal dialysis-associated peritonitis caused by *Eikenella corrodens* combined with *Enterococcus avium*: A case report and literature review

**DOI:** 10.1097/MD.0000000000047246

**Published:** 2026-01-30

**Authors:** Jia Guo, Lin Zhu, Jieyu Zhao, Yi Li, Yingye Miao

**Affiliations:** a Department of Nephrology, Guiqian International Hospital, Gui Yang, Guizhou, China; b Department of Nephrology, First Affiliated Hospital, Third Military Medical University (Army Medical University), China; c Department of Clinical Laboratory Medicine, Guiqian International Hospital, Gui Yang, Guizhou, China.

**Keywords:** case report, *Eikenella corrodens*, *Enterococcus avium*, peritoneal dialysis, peritonitis

## Abstract

**Rationale:**

Peritoneal dialysis-associated peritonitis (PDAP), caused by co-infection with *Eikenella corrodens* and *Enterococcus avium* is extremely rare. To the best of our knowledge, this is the first reported case of successful management without catheter removal.

**Patient concerns:**

A 75-year-old male on continuous ambulatory peritoneal dialysis presented with persistent abdominal pain, abdominal distension, and cloudy peritoneal dialysate.

**Diagnoses:**

Microbiological cultures of the peritoneal dialysate identified *Eikenella corrodens* and *Enterococcus avium* as causative pathogens.

**Interventions:**

The initial empirical treatment with intraperitoneal cefoperazone-sulbactam and cefazolin was ineffective. Based on the antimicrobial susceptibility results, the regimen was switched to levofloxacin, which targets both organisms.

**Outcomes:**

After a 3-week course of levofloxacin, the patient’s symptoms resolved completely, the peritoneal dialysate indices normalized, and the peritoneal catheter was successfully retained.

**Lessons:**

This case highlights that the early identification of atypical pathogens and targeted antibiotic therapy can lead to favorable outcomes in rare co-infections causing PDAP, potentially avoiding catheter removal.

## 1. Introduction

Peritoneal dialysis (PD)-associated peritonitis (PDAP) is a common complication among patients undergoing PD, with a mortality rate of 3% to 10%. Severe or recurrent episodes of PDAP are a leading cause of treatment failure, often resulting in a switch from PD to hemodialysis.^[1,2]^ *Eikenella corrodens* (*E. corrodens*), a gram-negative conditionally pathogenic bacterium that typically colonizes human mucosal surfaces and may lead to various infectious diseases, including periodontitis, parotiditis, liver abscesses, and brain abscesses under specific conditions.^[3]^ *Enterococcus avium* (*E. avium*) is a gram-positive conditionally pathogenic bacterium responsible for numerous severe hospital-acquired infections, such as urinary tract infections, endocarditis, and peritonitis.^[4,5]^ Currently, there are few reports of PDAP caused by *E. corrodens* and *E. avium* individually, and cases of PDAP involving *E. corrodens* in combination with other pathogens are even rarer. To date, there has been only 1 reported case of PDAP caused by *E. corrodens* combined with *Prevotella* species.^[6]^ Here, we describe the first reported case of PDAP caused by co-infection with *E. corrodens* and *E. avium*, successfully treated without catheter removal. We also reviewed the relevant literature on this rare co-infection.

## 2. Case description

A 75-year-old male patient was admitted to the hospital with a 48-hour history of abdominal pain accompanied by cloudy peritoneal dialysate. He was diagnosed with end-stage renal disease 4 years prior and subsequently underwent PD catheter placement. The patient had a medical history of hypertension, but no known history of chronic oral or gastrointestinal infections. He denied recent dental procedures or periodontal disease. Since then, he has been on continuous ambulatory peritoneal dialysis for more than 4 years. His current PD prescription included 1.5% dextrose dialysate: 2 L for 4 exchanges, with the final 2 L bag used for an overnight dwell. The patient’s ultrafiltration rate was approximately 600 to 700 mL/day, and his urine output was approximately 300 mL/day. During the 4 years of PD treatment, he had experienced 3 previous episodes of peritonitis. The first and second episodes, which occurred approximately 2 and 3 years after the initiation of PD, were both caused by *Staphylococcus epidermidis* and resolved after 14 days of intraperitoneal cefazolin therapy without catheter removal. The third episode occurred about 6 months before the current admission and was caused by *Escherichia coli*; the infection improved after treatment with intraperitoneal cefoperazone-sulbactam. All previous episodes were successfully treated, but repeated infections may have led to peritoneal fibrosis and impaired local immune function, thereby increasing the patient’s susceptibility to subsequent opportunistic infections.

On admission, the patient presented with paroxysmal colicky abdominal pain, accompanied by cloudy peritoneal dialysate without fever, nausea, vomiting, or diarrhea. Abdominal computed tomography was performed to exclude catheter malposition, intra-abdominal abscess, or bowel perforation, and no abnormal findings were detected. Laboratory tests showed a white blood cell count of 5.53 × 10^9^/L, neutrophil percentage of 76.10%, hemoglobin level of 85 g/L, C-reactive protein (CRP) of 12.08 mg/L, and albumin level of 27.18 g/L. Peritoneal dialysate analysis showed a total leukocyte count (TLC) of 960 × 10^6^/L, with a polymorphonuclear cell (PMN) percentage of 76.1%. Bacterial cultures and fungal pathogens were performed in the peritoneal dialysate. Following the 2022 guidelines from the International Society for PD,^[7]^ empirical treatment with intraperitoneal cefoperazone-sulbactam (1.5 g/qd) and cefazolin (1.0 g/qd) was initiated.

**Figure 1.**
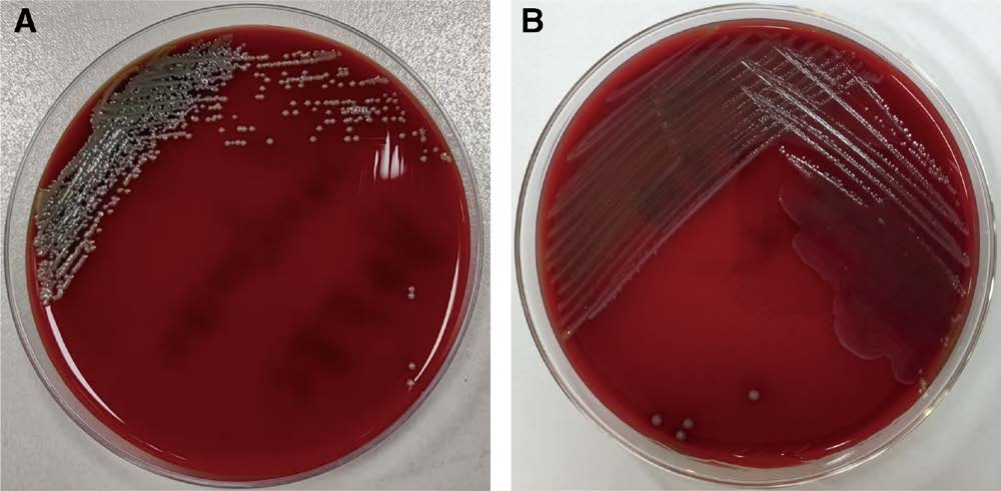
Growth characteristics of *Eikenella corrodens* and *Enterococcus avium* on blood agar. (A) *E. corrodens* forming characteristic pitted and dew-drop-like colonies on blood agar. No distinct hemolytic zones are observed. (B) *E. avium* forming characteristic gamma-hemolytic (nonhemolytic) colonies on blood agar, without clear or green hemolysis.

On day 4, the patient developed fever with worsening abdominal pain and cloudy peritoneal dialysate. TLC in the peritoneal dialysate increased to 6223 × 10^6^/L, and the proportion of PMN was 94%. The peritoneal dialysate was first incubated in blood culture bottles to improve the bacterial yield, followed by further culture on standard agar plates and selective media. On blood agar, *E. corrodens* forms small, translucent, or grayish-white colonies with smooth edges, some of which may be slightly depressed without distinct clear or green hemolytic zones (Fig. 1A). In contrast, *E. avium* forms grayish-white to milky-white colonies with well-defined margins and a moist surface without evident hemolysis (Fig. 1B). Antimicrobial susceptibility testing revealed that both *E. corrodens* and *E. avium* were susceptible to levofloxacin. Based on these results, the antibiotic regimen was adjusted, previous intraperitoneal antibiotics were discontinued, and the patient was started on oral levofloxacin 250 mg once daily.

On day 10, the patient’s clinical course was favorable with alleviation of abdominal pain, no signs of fever, and clear peritoneal dialysate. The TLC in the peritoneal dialysate significantly decreased to 810 × 10^6^/L, with the proportion of PMN dropping to 84.2%. Given the favorable clinical response, current treatment with oral levofloxacin 250 mg once daily was continued.

**Figure 2.**
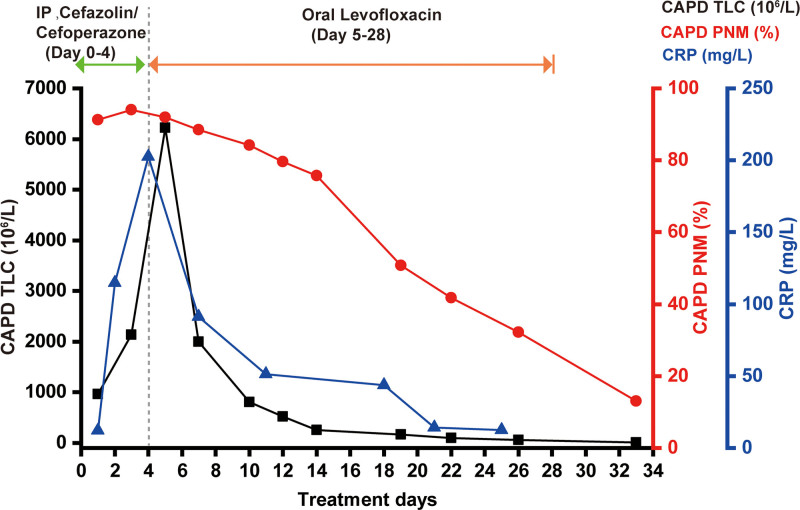
TLC and PMN percentage in the peritoneal dialysate, along with CRP levels, throughout the treatment period. CAPD = continuous ambulatory peritoneal dialysis, CRP = C-reactive protein, IP = intraperitoneal, PMN = polymorphonuclear cell, TLC = total leukocyte count.

After 26 days, the patient’s clinical symptoms showed significant improvement, with the alleviation of abdominal pain. Examination of the peritoneal dialysate revealed a TLC of 54 × 10^6^/L and a PMN proportion of 32.2%. The PD transfer set has been replaced. Two weeks after discharge, a peritoneal dialysate examination revealed a TLC of 6 × 10^6^/L, with a PMN proportion of 13%. The patient’s ultrafiltration rate returned to approximately 800 to 900 mL/day. During the follow-up period, no recurrence of peritonitis symptoms was observed, and the patient remains free of recurrence to date. No adverse events, such as drug-related allergic reactions, gastrointestinal discomfort, or catheter-related complications, occurred during the treatment or follow-up period. The dynamic changes in various inflammatory markers, including TLC and PMN percentage in the peritoneal dialysate, and CRP levels during the treatment period are shown in Figure 2.

The patient felt anxious at the onset of peritonitis due to his prior recurrent infections. After effective antimicrobial therapy, the infection was controlled without catheter removal, bringing relief and renewed confidence in continuing PD. He stated that this experience made him aware of the effects of long-term dialysis on peritoneal changes and immune decline, emphasizing the need for regular follow-up and timely medical care to prevent recurrence.

## 3. Discussion

*E. corrodens* is a member of the HACEK group (*Haemophilus*, *Aggregatibacter*, *Cardiobacterium*, *Eikenella*, and *Kingella*) and Neisseriaceae family. It is a facultatively anaerobic, nonmotile Gram-negative bacillus characterized by a slow growth rate and is the sole currently recognized species of the genus *Eikenella*.^[8]^ It typically colonizes the mucosal surfaces of the oral cavity, upper respiratory tract, digestive tract, and reproductive tract in humans.^[9]^ Although normally symbiotic, it can become a conditional pathogen in the presence of reduced immunity or impaired mucosal barriers, leading to various infections, including head and neck infections, endocarditis, pericarditis, lung infections, arthritis, brain abscesses, digestive infections, urinary tract infections, and skin and soft-tissue infections.^[10]^

*E. avium*, a member of the genus *Enterococcus*, is a Gram-positive bacterium that exists either aerobically or as a facultative anaerobe.^[11]^ It appears singly, in pairs, or in short chains. The genus *Enterococcus* includes 29 strains, among which *Enterococcus faecalis* and *Enterococcus faecium* are more prevalent, whereas *E. avium* is relatively rare. Found primarily in the intestinal microflora of birds, *E. avium* is not typically pathogenic in healthy individuals, but can be detected in the human gut. Nonetheless, it poses a risk in immunocompromised individuals, where it can cause hospital-acquired infections such as urinary tract infections, endocarditis, and abdominal infections. *E. avium* represents a significant challenge in infection control because of its natural resistance to a broad spectrum of antibiotics.^[12]^ Moreover, its ability to coexist with other hospital-acquired pathogens, either symbiotically or competitively, adds complexity to the disease and may affect therapeutic choices.^[13]^

Currently, there are no reports of PDAP caused by co-infection with *E. corrodens* or *E. avium*, both of which are opportunistic pathogens that cause infections under specific conditions. *E. corrodens* primarily colonizes the oral cavity and gastrointestinal tract, whereas *E. avium* may enter the body through the PD catheter system, suggesting that this case involved a multi-route co-infection. As *E. avium* is a common commensal bacterium in the intestines of birds, airborne transmission or hand contact may increase the risk of infection. Upon hospital admission, evaluation of the patient’s PD technique revealed nonadherence to aseptic protocols, including insufficient hand hygiene and prolonged exposure of the catheter connection, which could have led to PD catheter contamination and an increased risk of peritonitis. Additionally, the patient had a history of 3 previous episodes of peritonitis, which may have resulted in peritoneal fibrosis, impairing peritoneal permeability, and local immune barrier function, thereby increasing susceptibility to opportunistic infections. The combination of chronic inflammation, immunocompromised status, and improper PD procedures may have facilitated co-infection with *E. corrodens* and *E. avium*, thus complicating the clinical course and therapeutic management. These findings highlight that in PDAP, host-related factors, environmental exposure, and inadequate catheter care may collectively contribute to the emergence of rare co-infections, underscoring the need for stringent infection control measures in patients with PD. Millán et al described a case of PDAP in a 50-year-old patient who developed the condition posttransplant, caused by a mixed infection with *E. corrodens* and *Prevotella*. This complication likely resulted from the long-term use of immunosuppressants posttransplantation, which further weakened the patient’s immune defenses, indicating that *E. corrodens* can co-infect other bacteria, resulting in PDAP.

*E. corrodens* is susceptible to β-lactams, fluoroquinolones, and tetracyclines, but exhibits inherent resistance to clindamycin, metronidazole, and macrolides, and is less susceptible to erythromycin and aminoglycoside antibiotics.^[14]^ Millán et al also reported a case of PDAP caused by co-infection with *E. corrodens* and *Prevotella*, treated initially with cefradine and tobramycin.^[6]^ Following the drug sensitivity results from the bacterial culture of peritoneal dialysate, treatment was switched from cephalosporin to aminoglycoside antibiotics. The patient initially improved, but developed abdominal pain a week later, accompanied by cloudy peritoneal dialysate and a white cell count of 234 × 10^6^/L in the dialysate. Subsequent culture revealed the growth of *Prevotella oralis* resistant to penicillin and *Enterococcus faecalis*. Subsequently, all previous antibiotics were discontinued and intraperitoneal imipenem was initiated at a dose of 500 mg, followed by 200 mg per exchange for 15 days. The patient’s symptoms were completely alleviated without the need for removal of the PD catheter. Sheng et al reported that *E. corrodens* is susceptible to penicillin, amoxicillin, cefoxitin, cefotaxime, cefepime, ciprofloxacin, and imipenem, but is more prone to resistance to clindamycin, metronidazole, cephalothin, and cefuroxime.^[15]^ Infections caused by *E. corrodens* are prone to recurrence, especially when antimicrobial treatment is inadequate. Therefore, a sufficiently long treatment course of at least 3 weeks is recommended.

PDAP caused by *E. avium* is also rare, with only 5 cases reported to date. In a single-center retrospective study of 1421 cases of peritonitis in Hong Kong, enterococci were identified in 29 cases (2%), of which only 1 was attributed to *E. avium*.^[16]^ Peritonitis caused by *E. avium* may be due to intestinal translocation or urinary tract infections, as *E. avium* typically colonizes these systems.^[17]^ Moreover, most reported cases of PDAP caused by *E. avium* are associated with a history of gastrointestinal tract disease. Calça et al reported the case of a 37-year-old male patient with PDAP caused by *E. avium*. The bacterium was found to be sensitive to ampicillin, vancomycin, and gentamicin. The patient was treated with intravenous ampicillin (1000 mg, q6h) and gentamicin (0.6 mg/kg, q48h), resulting in significant clinical improvement. After discharge, the patient continued intravenous vancomycin and gentamicin, completing a 21-day course of antibiotics.^[18]^

A review of the existing literature identified several previously reported cases of PDAP caused by *E. corrodens* or *E. avium*, which are summarized in Table 1. Most of these infections were caused by a single pathogen, although a few involved mixed infections in which *E. corrodens* coexisted with other microorganisms such as *Prevotella* species.^[19,20]^ The present case represents the first documented PDAP case caused by co-infection with *E. corrodens* and *E. avium*. *E. corrodens* has biofilm-forming capabilities, allowing it to adhere to the surface of PD catheters, leading to chronic infections or persistence of bacterial aggregates that are difficult to eradicate. *E. avium* exhibits strong adhesion to host cells and can survive within biofilms, further enhancing the persistence of the infection. These 2 bacteria may interact to increase their virulence, resulting in more severe inflammatory responses and rapid disease progression. The initial empirical antibiotic therapy was ineffective, as evidenced by a rapid increase in the peritoneal dialysate leukocyte count and worsening clinical symptoms, including fever and exacerbated abdominal pain on day 4. The clinical presentation in this patient differed from that of typical PDAP caused by a single pathogen, as the early symptoms were relatively mild, but the disease progressed rapidly. This may be related to the synergistic pathogenic effects of *E. corrodens* and *E. avium*. *E. corrodens* is resistant to various β-lactam antibiotics, particularly cephalosporins, making the empirical use of cephalosporin ineffective. However, antimicrobial susceptibility testing revealed that both organisms were sensitive to levofloxacin. After the antibiotic regimen was adjusted, the patient was treated with oral levofloxacin 250 mg once daily, according to the 2022 International Society for PD guidelines,^[7]^ which recommend an oral dose of 250 mg daily or 500 mg every 48 hours. The patient’s symptoms significantly improved following this adjustment. On day 14, the white blood cell count in the peritoneal dialysate decreased to 164 × 10^6^/L, and the same oral dose of levofloxacin (250 mg once daily) was continued without change. After 28 days, the patient’s symptoms had resolved completely. The treatment of mixed infections caused by *E. corrodens* and *E. avium* poses significant challenges, primarily due to *E. corrodens* to cephalosporins, which leads to the failure of empirical antibiotic therapy. The ability of E. avium to survive within biofilms increases the persistence of the infection, potentially requiring an extended treatment duration. The standard dose of intraperitoneal levofloxacin for treating PDAP remains unclear and necessitates pharmacokinetic adjustments to optimize its efficacy while minimizing adverse effects. Therefore, for co-infections with *E. corrodens* and *E. avium*, prompt and appropriate management is crucial to prevent recurrence, particularly in immunocompromised patients. It is recommended that treatment be guided by drug sensitivity testing and to ensure continuous antibiotic administration for at least 3 weeks to achieve the best outcomes.

**Table 1:** Summary of published cases of *Eikenella corrodens* or *Enterococcus avium*-related peritonitis in peritoneal dialysis.

Study	Age/sex	Dialysis vintage	Peritoneal culture organism	Antibiotic used/route	Outcome
Adapa et al^[19]^	60/M	2 yr	*Enterococcus avium*	Vancomycin/IP → Linezolid/Oral	PD catheter removed, switched to HD
Chao et al^[17]^	79/F	>2 yr	*Enterococcus avium*	Cefazolin + Ceftazidime/IP→ Ampicillin/IP	PD catheter preserved
Calça et al^[18]^	37/M	5 yr	*Enterococcus avium*	Cefazolin + Ceftazidime/IP →Cefazolin + Gentamicin/IP + Ciprofloxacin/Oral →Vancomycin + Gentamicin/IV	Catheter removed →temporary HD→PD resumed →kidney transplant
Millán Díaz et al^[6]^	50/M	9 yr	*Eikenella corrodens,* *Prevotella oralis,* *Enterococcus faecalis,* *Escherichia coli,* *Bacteroides merdae*	Cefazolin + Tobramycin/IP →Vancomycin/IP →+Metronidazole/IP →Imipenem/IP	PD catheter preserved
Ugur et al^[20]^	10/F	2 yr	*Enterococcus avium*	Amikacin/IP →+Vancomycin/IP	Peritonitis cured, but patient died due to intracranial hemorrhage unrelated to infection

HD = hemodialysis, IP = intraperitoneal, IV = intravenous, PD = peritoneal dialysis.

## 4. Conclusion

In summary, PDAP caused by co-infection with *E. corrodens* and *E. avium* is rare. Appropriate antibiotics should be selected based on drug sensitivity testing and treatment efficacy must be closely monitored. Long-term follow-up is essential for managing potential recurrence. Culturing *E. corrodens* is challenging because of its stringent requirements, which make it difficult to grow. Therefore, clinicians must remain vigilant, even when peritoneal dialysate cultures are negative.

## Author contributions

**Conceptualization:** Jia Guo, Yi Li, Yingye Miao.

**Data curation:** Lin Zhu, Jieyu Zhao.

**Writing – original draft:** Jia Guo, Yi Li.

**Writing – review & editing:** Lin Zhu, Jieyu Zhao, Yingye Miao.
